# ncRNAs-mediated overexpression of STIL predict unfavorable prognosis and correlated with the efficacy of immunotherapy of hepatocellular carcinoma

**DOI:** 10.1186/s12935-023-02869-y

**Published:** 2023-03-10

**Authors:** Longwen Xu, Shirong Zhang, Jinteng Feng, Deli Tan, Hong Sun, Hui Guo

**Affiliations:** Department of Medical Oncology, The First Affiliated Hospital of Xi’an Jiaotong University, Xi’an, 710061 People’s Republic of China

**Keywords:** STIL centriolar assembly protein (STIL), Prognosis, Non-coding RNAs (ncRNAs), Tumor-infiltrating immune cell, Immunotherapy

## Abstract

**Background:**

STIL centriolar assembly protein (STIL) is a cytoplasmic protein implicated in cellular growth and proliferation as well as chromosomal stability, which abnormal condition affected tumor immunity and tumor progression. However, the role of STIL in the biological mechanism of hepatocellular carcinoma (HCC) remains unclear.

**Methods:**

Comprehensive bioinformatic approaches, in vitro functional assays, and validation were conducted to elucidate the oncogenic value of STIL in HCC.

**Results:**

In the present study, we found that STIL may serve as an independent prognostic indicator and a potential oncogene in HCC. Gene set enrichment analysis (GSEA), and Gene set variation analysis (GSVA) showed that upregulated expression of STIL was positively associated with pathways enriched in the cell cycle and DNA damage response. Subsequently, we identified several non-coding RNAs (ncRNAs) accounting for the upregulation of STIL expression using a combination of in silico bioinformatics approaches (including expression analysis, correlation analysis, and survival analysis). Finally, CCNT2-AS1/SNHG1-has-miR-204-5p-STIL axis was screened out as the most potential upstream ncRNA-related pathway of STIL in HCC. Moreover, STIL expression is highly associated with the infiltration of immune cells, the expression of immune checkpoints, as well as the survival benefit of immunotherapy/chemotherapy.

**Conclusions:**

Our study discloses that ncRNAs-mediated overexpression of STIL independently predicted poor prognosis and correlated with the efficacy of PD-1-targeted immunotherapy in HCC.

**Supplementary Information:**

The online version contains supplementary material available at 10.1186/s12935-023-02869-y.

## Introduction

Hepatocellular carcinoma (HCC), a significant type of primary liver cancer, is the third most common cause of cancer-related death worldwide, usually with poor clinical outcomes [1–4]. Amounts of risk factors are linked to either the induction of HCC or its progression. These factors included HBV or HCV infection, cirrhosis or advanced fibrosis, alcohol abuse, nonalcoholic fatty liver disease, and so on. Besides, several signaling pathways (such as PD1/PD-L1, TGF-β, Notch, HGF, VEFG, and HIF) have been proven to be dysregulated in HCC and drive uncontrolled cell proliferation and metastasis [5–7]. Targeting specific signaling pathways could pave the way for promising treatment approaches to HCC [8]. Recently, mounting studies have confirmed that several immune checkpoint inhibitors (ICIs), including targeting PD-L1 inhibitors (atezolizumab), CTLA-4 inhibitors (nivolumab), and PD-1 inhibitors (pembrolizumab), are revolutionizing the treatment approaches in HCC by leading to increased survival benefits and long-lasting antitumor responses. However, only a minority of patients obtain a durable clinical benefit from ICIs. Hence, it is critical to identify a biomarker to predict clinical outcomes and the efficacy of ICIs for HCC.

The tumor microenvironment (TME) is defined as a complex mixture of cellular and noncellular components surrounding tumor mass that exerts a crucial role in cancer cell growth, invasion, migration and metastasis among multiple malignancies, including HCC [9]. Of the cellular components, immune cells within the TME have been demonstrated to be participate in the development of HCC and in the response to cancer immunotherapy. Mounting evidences have indicated the interaction between genomic instability and TIME. For instance, previous studies revealed that genomic instability may activate CD4+ T cells and increase T-cell cytolytic activity through the production of neoantigens [10, 11]. DNA damage response alterations, fueled by either cytotoxic agents or deletion of normal DNA repair ability, may contribute to antitumor immunity in cGAS/STING pathway, recently emerging as an essential mechanism to trigger inflammation-driven tumor growth [11–13]. In addition, high levels of somatic copy-number alterations (gain or loss), triggered by chromosome Instability, a major mode of genomic instability, have closely link with reduced expression of cytotoxic immune cell markers, leading to immune suppression [14]. Therefore, preventing chromosome Instability (CIN) may be a breakthrough point to sensitize tumors to immunotherapy. Chromosome Instability has been identified as one of the major modes of genomic instability.

The STIL Centriolar Assembly Protein (STIL) was first discovered as a deleted locus in T-cell acute lymphoblastic leukemia. STIL gene was found to be a multifunctional protein participating in diverse cellular functions, including chromosomal stability and cellular proliferation. For instance, STIL exerts a functional role in maintaining centrosome integrity through encoding a centriolar protein required for centriole replication [15–22]. Overexpression of STIL leads to supernumerary centrosomes, which were linked to cancer-causing chromosomal instability (CIN) [21, 23]. Furthermore, CIN cancers are reported to possess T cell exclusion and infiltrating macrophages, and the centrosome is critical for cytokine production, which is necessary for the immune response, suggesting that STIL is probably involved in the tumor immune microenvironment [24]. Besides, increasing studies suggest that STIL has been found significantly upregulated in multiple malignancies, including lung cancer, ovarian cancer, prostate adenocarcinoma, and gastric cancer [15, 25, 26]. Meanwhile, STIL expression is linked with high metastatic capacity in adenocarcinoma [27]. However, the relationship between STIL and tumor immunity and its expression pattern, clinical outcomes, and potential regulatory pathway in HCC progression remains determined.

The ncRNAs represent a type of non-coding RNAs (including lncRNA, miRNA, circRNA, etc.) that do not encode proteins, and participate in novel patterns of regulating gene expression. These ncRNAs play an important role in the proliferation, invasion, metastasis and drug resistance of cancer cells [28–30]. miRNAs are small ncRNAs, comprising about 22 nucleotides, and can impede the expression of target genes by interacting with their 3′UTR region. lncRNAs represent a class of ncRNA over 200 nucleotides in length, and can positively or negatively modulate gene expression through a range of ways. One theory that illustrates the mechanism of lncRNA and miRNA is the competitive endogenous RNAs (ceRNAs) hypothesis. This hypothesis proposes that lncRNA function as a molecular sponge of miRNAs to impede their expression, thereby lessening the inhibitory effect of miRNA on target genes [15, 31, 32]. Mounting evidence has confirmed this lncRNA-miRNA-mRNA regulatory network has been confirmed in various human cancers [33].

Thus, in this study, we comprehensively investigated the expression of STIL in multi-cohorts, and explored the relationship of STIL expression level with clinicopathological factors and clinical outcomes of HCC patients. Next, the ceRNA regulatory pathway of STIL was also examined in HCC. Finally, we analyzed the association of STIL expression with diverse immune-infiltrating cells, immune checkpoints, and the survival benefit of immunotherapy/chemotherapy in HCC. In sum, our results indicated that ncRNAs-mediated overexpression of STIL predicts poor prognosis and is associated with therapeutic efficacy in HCC patients.

## Methods

### HCCDB database analysis

The HCCDB database (http://lifeome.net/database/hccdb/home.html) comprises multiple HCC expression atlas containing 3917 samples from15 online cohorts of HCC gene expression, including 13 microarray cohorts from the GEO (Gene Expression Omnibus), two RNA sequencing (RNA-seq) cohorts from TCGA-LIHC (Liver Hepatocellular Carcinoma Project of The Cancer Genome Atlas) and ICGC-LIRI-JP (Liver Cancer RIKEN, JP Project from International Cancer Genome Consortium). HCCDB, as a user-friendly one-stop online dataset, provides the visualization for the investigation of HCC expression analysis [34].

### Oncomine database analysis

The mRNA expression data of the STIL gene in liver cancers was investigated in the Oncomine database (https://www.oncomine.org/resource/login.html) [35]. The threshold was set according to the following criteria: *P*-value of 0.001, fold change of 2.0, and gene ranking of all.

### RNA-seq data download and preprocessing

The expression data of messenger RNA (mRNA), non-coding RNA (ncRNA), and microRNA (miRNA) isoforms of HCC samples and clinical characteristics of matched samples were obtained from TCGA (The Cancer Genome Atlas) data portal (https://portal.gdc.cancer.gov/). The mRNA expression data and clinical characteristics of the ICGC-LIRI-JP dataset (n = 232) were abstracted from the ICGC (International Cancer Genome Consortium) dataset (https://dcc.icgc.org) and an immunotherapy cohort (IMvigor210) were retrieved from a previous study [36]. The raw RNA-seq transcriptome count data from TCGA, ICGC, and IMvigor210 were further transferred into a transcript per kilobase mullion (TPM), which is more similar to those resulting from microarrays and more comparable between samples [37]. We then conducted TCGA-LIHC survival information processing to selected cases with sufficient data. The best cut-off value of STIL expression was counted using receiver operating characteristic (ROC) analysis with the aid of the “survminer” R package (version 0.4.6). We compared the overall survival (OS) of HCC patients divided by the STIL cut-off expression value in the datasets of TCGA-LIHC, ICGC-LIRI-JP, and IMvigor210 [36]. Two hundred and twenty-two patients with complete clinical data were used for Cox regression analysis to investigate the relationship of clinicopathological factors and STIL expression level with OS in the TCGA-LIHC cohort. As the data (TCGA, ICGC, and IMvigor210 datasets) are publicly available, the patients involved in the database have obtained ethical approval, thus no ethical approval is required. R (https://cran.r-project.org/) (R software, version 3.6.3) and Strawberry Perl (version 5.32.1.1) were used for all preprocessing processes.

### Single-cell analysis

The detailed gene alteration and distribution information could be abstracted from single-cell data. The processed single-cell data containing 71,915 cells were obtained from the GSE149614 dataset [38]). The Seurat R software package was applied to reduce the dimensions of cells and generate a t-SNE (t-distributed stochastic neighbor embedding) diagram for clusters and cell-types visualization. In order to study the role of STIL in tumors, the comparison of STIL between different tissue sources and cell types was conducted.

### Gene mutation and heterogeneity

The MAF (Mutation Annotation Format) Profiles of simple nucleotide variation from the TCGA-LIHC cohort were abstracted from the TCGA GDC portal (https://tcga-data.nci.nih.gov/tcga/), which was processed using the workflow type of varScan2 variant aggregation and masking and visualized with the aid of R "maftools (version 2.14.0)" package. Tumor Mutation Burden (TMB) was calculated as the number of mutation base in per million bases based on somatic mutation data. The mutant-allele tumor heterogeneity (MATH) score is a simple quantitative measure of intra-tumor heterogeneity, which calculates the width of the vaf (variant allele frequency) distribution. Higher MATH scores indicate the higher heterogeneity, and tend to be associated with worse clinical outcomes. TMB and MATH score of tumor tissues in the TCGA-LIHC cohort were also counted via the "maftools" package.

### TIMER analysis

The TIMER (Tumor Immune Estimation Resource) database is a comprehensive resource for systematically detecting the abundance of immune-infiltrating cells among 32 cancer types using more than ten thousand samples from TCGA (https://cistrome.shinyapps.io/timer/) database [39]. TIMER applies a deconvolution approach to determine the infiltration of six immune cells relying on the mRNA expression profiles [40]. The relationship between the expression level of STIL gene and the abundance of six immune infiltrates, including CD4 + T cells, CD8 + T cells, B cells, neutrophils, dendritic cells, and macrophages, as well as the tumor purity. The marker genes used for the analysis of multiple immune infiltrates, including T cells, B cells, monocytes, TAMs, macrophages M1, macrophages M2, natural killer cells, dendritic cells, neutrophils, T-helper cells, and Tregs were relied on previous studies [41, 42].

### Gene set enrichment analysis (GSEA) and Gene set variation analysis (GSVA)

GSEA and GSVA were used to investigate the mechanisms of STIL in HCC. In this study, the enrichment scores (ES) of "c2.cp.kegg.v6.2.symbols.gmt" gene sets from the MSigDB (Molecular Signatures Database) in each group were counted by GSEA software (version 4.1.0) and reflected the degree to which a given gene set is represented in a ranked list of genes [43, 44]. A nominal P-value of < 0.05 and an FDR q-value of < 0.25 were used as the cut-off criteria. Besides, GSVA was conducted to further explore the disparity of pathways between the phenotypes of STIL performed by the “GSVA” R package (version 1.40.1) [45].

### Single-sample gene set enrichment analysis (ssGSEA)

The enrichment score of sixteen infiltrating immune cells, as well as thirteen immune-related functions or pathways, were performed by the GSVA package with a single-sample gene set enrichment method, which can help judge the activity of immune cells, immune function, or immune pathway of each sample.

### Construction of a competing endogenous RNAs (ceRNA) network of STIL

The dysregulated expression of miRNAs exerts a pivotal role in regulating target gene expression. To further screen the interacting miRNAs of STIL, the associated miRNAs were first predicted from the encyclopedia of RNA interactomes (ENCORI, http://starbase.sysu.edu.cn/), and then the most potential interacting miRNAs of STIL were selected by the "limma" R package (version 3.48.3) according to the following analysis: differential analysis (|fold change|> 1, *P*-value < 0.001), correlation analysis (spearman's correlation coefficient > 0.1) and survival analysis (log-rank* P*-value < 0.05). Next, the selected miRNAs were used to search associated lncRNAs from ENCORI. The predicted lncRNAs were further analyzed for the differential expression (*P*-value < 0.001 and absolute fold change > 1), and correlations with prognosis and STIL (spearman’s correlation coefficient > 0.1 and log-rank* P*-value < 0.05). Last, we constructed a ceRNA (competitive endogenous RNA) network, which refers to the lncRNA-miRNA-mRNA regulatory network, relying on the relationship of miRNAs, STIL, and lncRNAs.

### Evaluation of STIL protein by immunohistochemistry (IHC)

Tissue microarray (TMA) (including 92 pairs of HCC tissues and adjacent normal tissues) was obtained from OUTDO Biotech (Shanghai, China). IHC was conducted as described previously [46]. The primary antibody for STIL (human, diluted 1:200) was purchased from (Proteintech Group, Wuhan, China). In tumor cells, cytosol or nuclei that stained dark brown were considered positive for STIL, and faintly stained cytosol or nuclei were considered negative. The staining intensity was assessed with the following scoring system: 0 point (negative staining); 1 point (weak staining); 2 points (moderate staining); and 3 points (strong staining). Additionally, the percentage of stained tumor cells was evaluated as follows: 0 point (0%); 1 point (less than 25%); 2 points (25–50%); and 3 points (more than 50%). the above two scores were multiplied to obtain the final score. The final score ranging between 0 and 3 points defined a low expression level of STIL, while the final score greater than 3 points represented high expression.

### Cell culture, RNA Interference, and western blotting analysis

Human liver cancer cells (Hep3B and Huh7) were a gift from JuSeog Lee (MD Anderson Cancer Center, Houston, TX). All cells were cultured in high glucose Dulbecco’s modified Eagle’s medium (Hyclone, Logan, UT) supplemented with 10% FBS (Hyclone, Logan, UT) and 100 units/mL penicillin and streptomycin. Cell lines were incubated with a humidified atmosphere incubator of 5% CO2 at 37 °C. The human STIL-target small interfering (si) RNAs (siRNA1: 5′-3′ CGCCAGUAUUGAGAAAUAUTT, 3′-5′ AUAUUUCUCAAUACUGGCGTT; siRNA2: 5′-3′ GCACCUUCUUAGAUGUAAATT, 3′-5′ UUUACAUCUAAGAAGGUGCTT; siRNA3: 5′-3′ GCAGUGAUCUCUGGAUUAATT, 3′-5′ UUAAUCCAGAGAUCACUGCTT) were utilized to establish STIL knockdown HCC lines, by the aid of Lipofectamine 2000 (Invitrogen, MA). Then Western blot was used to evaluate the knockdown effect of these siRNAs on HCC lines, according to standard protocols. The following primary antibodies were applied: anti-GAPDH antibody (human, diluted 1:20,000, 10494-1-AP, Proteintech Group, Wuhan) and anti-STIL antibody (diluted 1:2000, Proteintech Group, Wuhan).

### Immunofluorescence staining

Transfected cells grown on glass coverslips were fixed with 4% paraformaldehyde for 10 min at room temperature. Subsequently, the cells were washed with PBS, permeabilized with Triton X-100 at a concentration of 0.2% for 5 min, and then blocked for 1 h with 1% BSA solution in PBS. These cells were incubated with primary monoclonal antibodies against STIL (1:100 dilution, Santa Cruz, China) at 4 °C overnight. The next day, the coverslips were incubated for 2 h in a dark room with TRITC-labeled anti-mouse secondary antibody (1:200 dilution, Southern Biotech), then the nuclei were stained with DAPI for 5 min at 4 °C. Finally, LSM80 fluorescent microscope was applied to observe the STIL expression in cells.

### Proliferation assay

CCK-8 and colony formation assays were conducted to investigate the effect of STIL silencing on the proliferation of Hep3B and Huh7 cells. Briefly, Hep3B/siRNA-con, Hep3B/siRNA-STIL, Huh7/siRNA-con, and Huh7/siRNA-STIL cells at a density of 2000/well were cultured in 100 μL of DMEM containing 10% FBS at 96-well plates for 3 days. Every day after culture, each plate of cells was added with 10 μl of CCK solution and incubated for 3 h at 37 °C, and then measured for the absorbance value (OD) at 450 nm in a microplate reader. As for the colony formation assay, these cells were seeded in 6-well culture plates at 1000 cells/well. After being cultured for 2 weeks at 37 °C, the cells were washed twice with PBS and stained with 0.1% crystal violet solution. The number of colonies containing 50 cells was computed. The colony formation efficiency was calculated as (colon numbers/inoculated 1000 cells) × 100%.

### Wound-healing assay

For the wound-healing assay, Hep3B/siRNA-con, Hep3B/siRNA-STIL, Huh7/siRNA-con, and Huh7/siRNA-STIL cells were plated in a six-well dish with serum-free medium. When the confluence of cells reaches 80%, an artificial wound track was performed by scraping the surface of the well with a pipette tip. The scratches were assessed every 24 h.

### Transwell assay

Transwell assay (including cell migration and invasion assays) was performed to assess cell motility by using transwell chambers with or without Matrigel (BD, Biosciences, CA). Approximately 3 × 10^4^ cells in FBS-free medium were cultured in upper transwell chambers with or without Matrigel and incubated at 37 °C for 24 h. Medium containing 10% FBS was put in the lower chamber. The invasive cells attached to the lower surface of the membrane insert were fixed, stained using Giemsa (Jiancheng, Jiangsu, China), and quantified under a microscope.

### Prediction of chemotherapeutic drug sensitivity between STIL-high and STIL-low subsets

The pRRophetic package, obtained from https://github.com/paulgeeleher/pRRophetic, was utilized to predict the half-maximal inhibitory concentration (IC50), an indicator of the sensitivity of drugs to tumor cells, of chemotherapeutic drugs (including cisplatin, paclitaxel, gemcitabine, doxorubicin, sorafenib, and sunitinib) for each patient between the STIL-high and STIL-low subsets.

### Statistical analysis

Software R (version 3.6.3) was applied to all data analyses. The Wilcoxon signed-rank test was employed to examine the relationship between clinical parameters and STIL. Spearman’s correlation analysis was employed to explore the relationship of gene expression in the TIMER database. Survival analysis was utilized by the Kaplan–Meier (KM) log-rank test. The comparison of scores of immune cells or pathways between the high- and low-STIL groups was explored by Mann–Whitney test. Independent indicators for overall survival were selected after employing univariate and multivariate Cox regression analyses. *P* values < 0.05 were considered statistically significant.

## Result

### STIL is overexpressed in HCC

**Fig.1:**
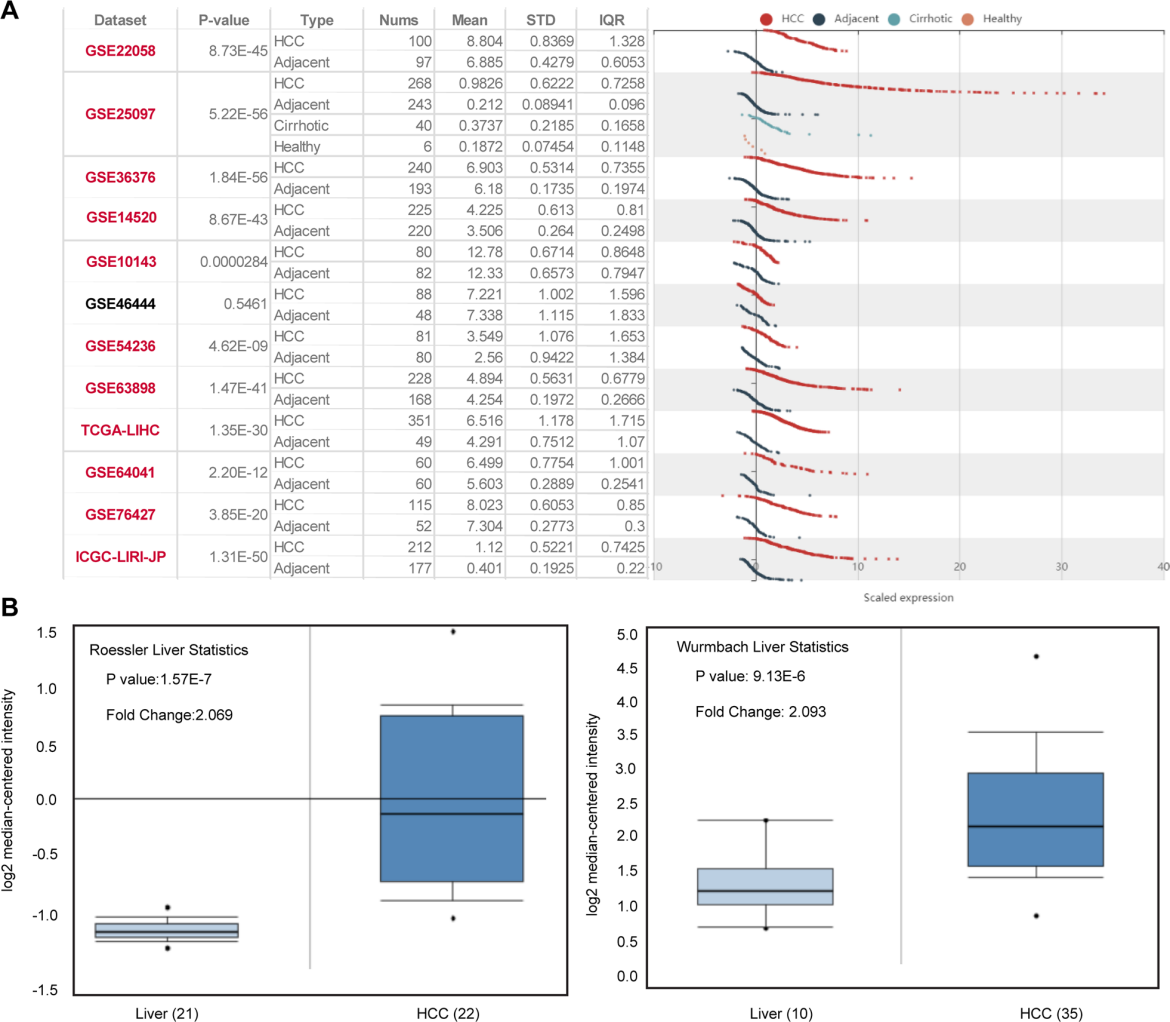
The STIL expression in HCC.** A** Chart and scatter showing the transcription level of STIL in HCC and the adjacent normal tissues in HCCDB. **B** Box plot showing STIL mRNA expression in the Roessler Liver, and Wurmbach Liver datasets in Oncomine database, respectively

To probe the potential roles of STIL in carcinogenesis, we investigated the STIL mRNA expression in HCC expression profiles from TCGA and GEO databases. Analysis of twelve HCC sets from the HCCDB database showed that the transcriptional level of STIL was remarkably upregulated in HCC tissues compared with normal adjacent tissues in eleven HCC cohorts (Fig. 1A). Similarly, the expression of STIL in HCC tissues exhibited an obviously higher level when compared with that in the normal adjacent tissues, in two different statistics from Oncomine (Fig. 1B). These results showed that STIL is significantly overexpressed in HCC tissues, implying that STIL may exert a crucial function in the oncogenesis of HCC.

### STIL expression serves as an independent prognostic indicator in HCC patients

**Fig. 2:**
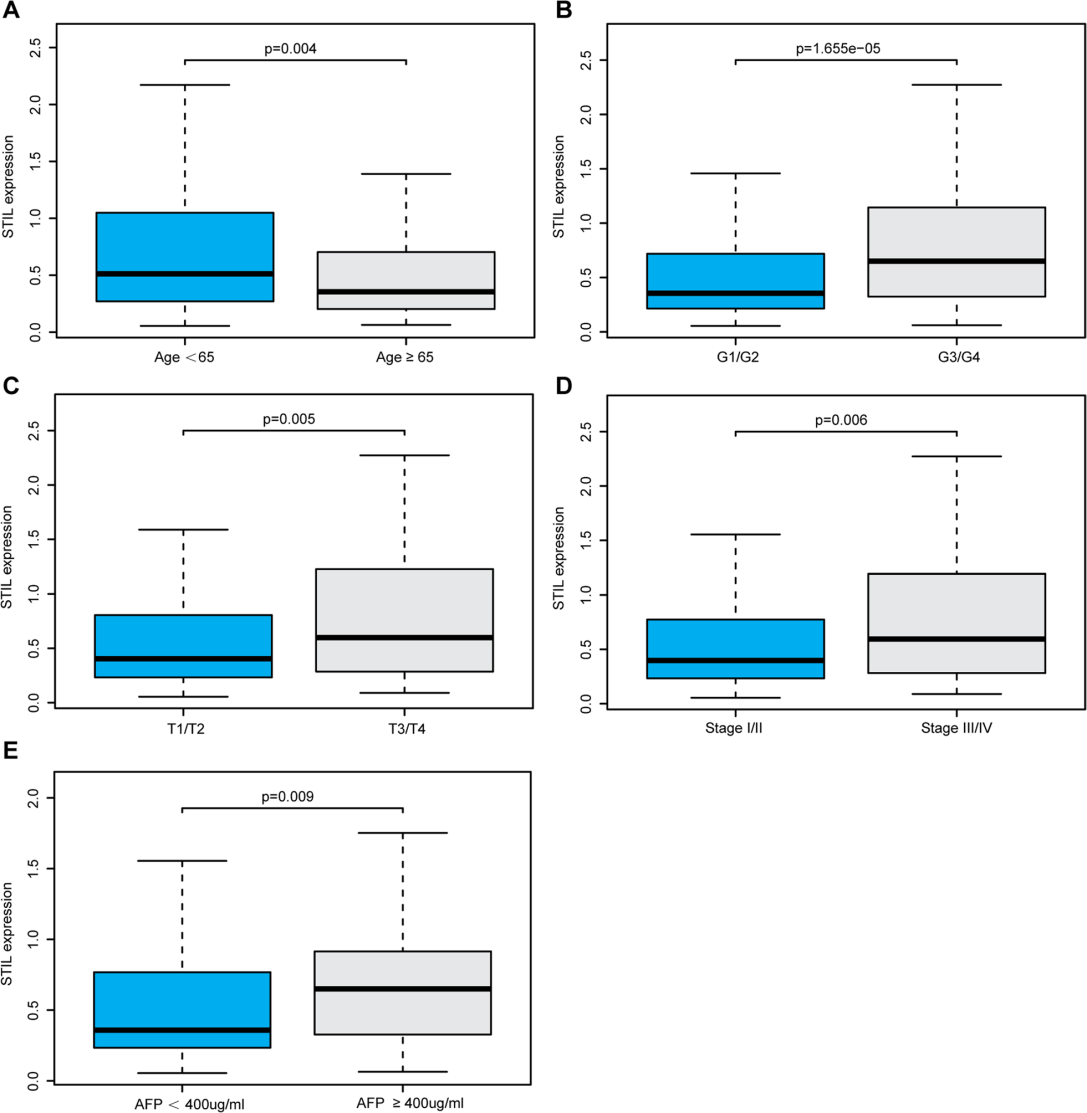
The expression of STIL and its relationships with clinicopathologic factors. The median value of STIL expression was set as the cut-off value. The Wilcoxon signed-rank test was applied to examine the association between clinicopathological varieties and the expression of STIL in 374 HCC samples. **A** Age, **B** histological grade, **C** T stage, **D** TNM stage, and **E** AFP levels. *STIL* STIL centriolar assembly protein; *HCC* hepatocellular carcinoma

**Fig. 3:**
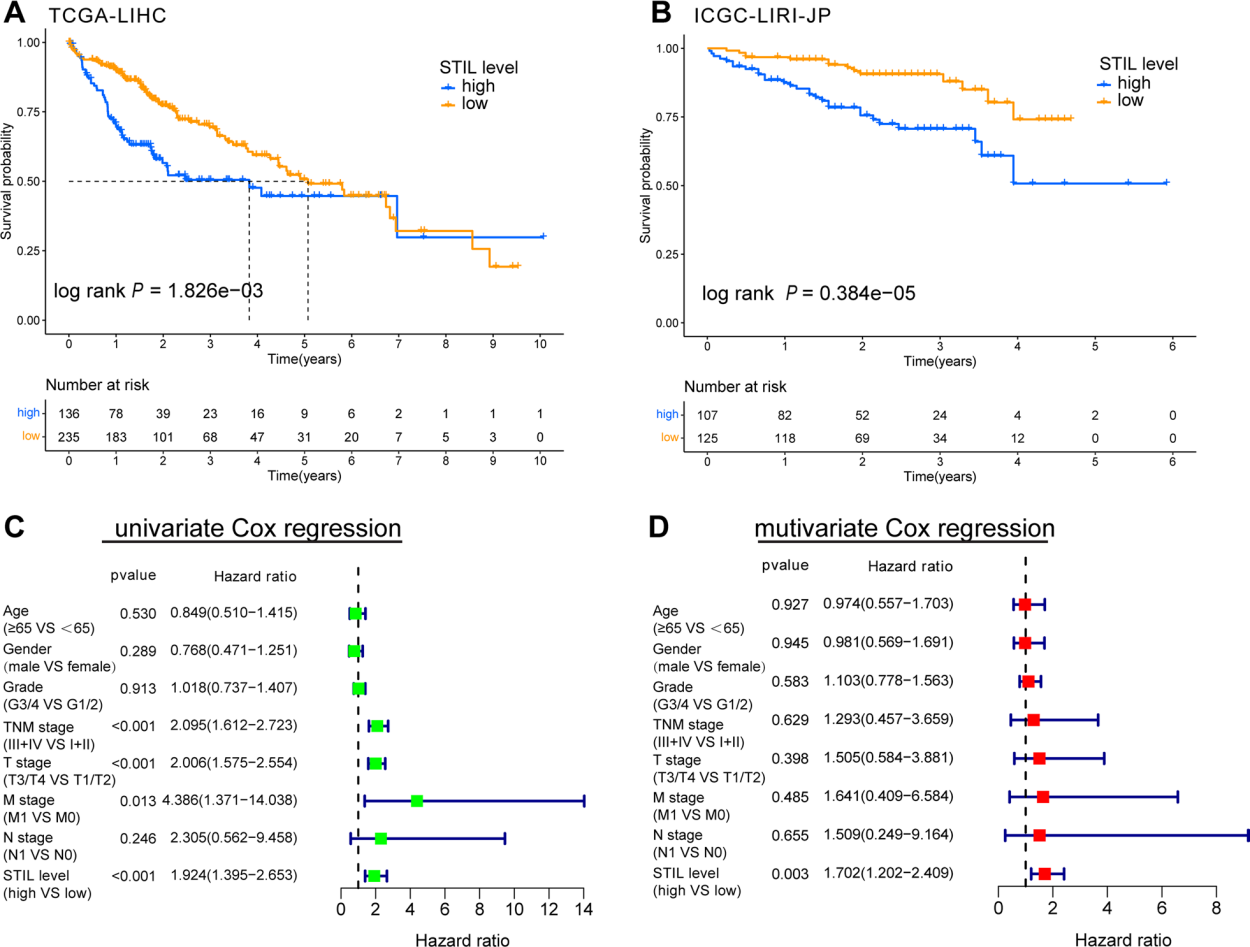
Prognostic significance of STIL expression in HCC. Kaplan–Meier survival curves showed that the overall survival (OS) of patients with HCC with high STIL expression was poor than that of patients with HCC with low STIL expression in **A** TCGA-LIHC and **B** ICGC-LIRI-JP cohort; Association with OS and clinicopathological factors in HCC patients from TCGA dataset using **C** univariate Cox regression **(D)** multivariate Cox regression

The observed overexpression of STIL initiated us to further explore the clinical significance of STIL in HCC. The potential relationships of STIL expression with some clinicopathological parameters were examined using the Wilcoxon signed-rank test. As shown in Fig. 2, the mRNA expression of STIL was significantly correlated with age (*P* = 0.004), grade (*P* = 1.655e−05), T stage (*P* = 0.005), TNM Stage (*P* = 0.006) and AFP (*P* = 0.009). However, there were no significant associations between STIL expression and other clinical factors, including gender, N stage, and M stage (data not shown). Furthermore, we compared the clinical outcome of STIL-high and STIL-low groups separated by the cut-off value of 4.264 among TCGA and ICGC datasets. In the LIHC cohort from TCGA, KM survival curves revealed that the STIL-high expression group had an unfavorable overall survival (OS) than the STIL-low expression group (*P* = 6.285e−04) (Fig. 3A). Likewise, the STIL-high expression group exhibited poorer outcomes (*P* = 0.384e−05) in the ICGC-LIRI-JP cohort from ICGC (Fig. 3B). Besides, the univariate Cox regression analysis revealed that high STIL expression was highly associated with poor clinical outcomes in TCGA cohort (hazard ratio [HR] = 1.826, 95% confidence interval CI [1.424–2.342], *P* < 0.001; Fig. 3C). Meanwhile, in multivariate analysis, only high expression of STIL (HR = 1.683, 95% CI [1.280–2.215],* P* < 0.001) still implied an inferior prognosis independent of other clinical parameters (Fig. 3D). Collectively, these results confirmed that STIL expression is an independent prognostic indicator and that high levels of STIL expression predict the poor OS of HCC.

### The expression of STIL was distinct in the tumor immune microenvironment

**Fig. 4:**
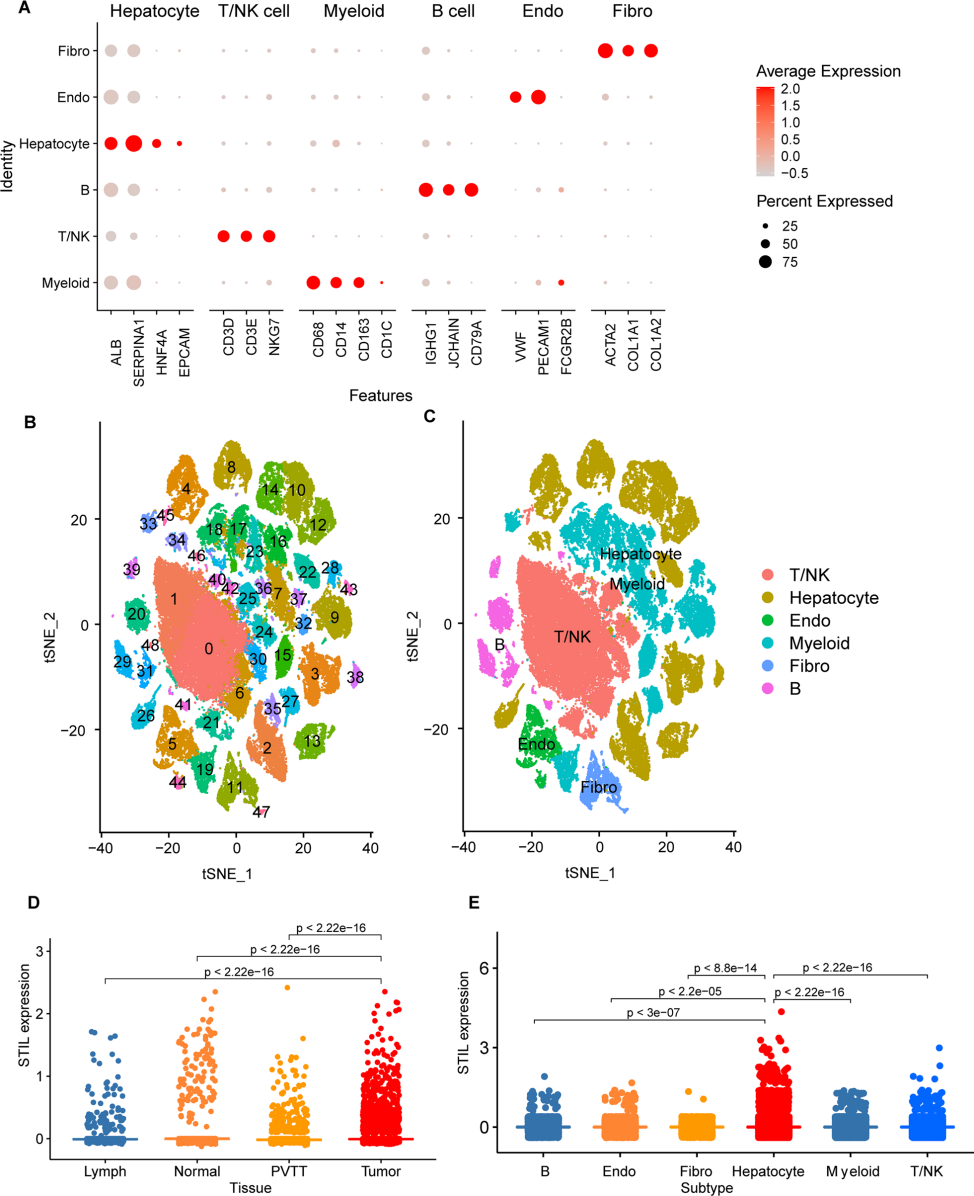
The expression of STIL in HCC by single-cell analysis. **A**, **B** By t-SNE analysis of HCC single-cell data, 71,915 cells were divided into 49 clusters and six cell types, including T and natural killer (NK) cell (T/NK), hepatocyte, endothelial cell (Endo), myeloid cell, fibroblast (Fibro), and B cell. **C**, **D** The scatter plot revealed the expression of STIL was distinct among different tissues and cell types

The scRNA-seq data of 71,915 cells from 10 HCC patients with 21 samples, including 10 samples from the primary tumor (PT), 2 samples from the portal vein tumor thrombus (PVTT), 8 samples from the non-tumor liver (NTL), and 1 sample from metastatic lymph node (MLN), was abstracted from GSE149614 dataset. A total of 49 cell clusters were identified using canonical gene markers of major cell populations and found they consist of 10 T and natural killer (NK) cell clusters,13 hepatocyte clusters, 2 endothelial cell clusters, 15 myeloid cell clusters, 2 fibroblast clusters, and 7 B cell clusters (Fig. 4A–C). We then compared the expression of STIL among different tissue sources and cell types. Consistent with the above result, STIL expression was significantly increased in primary tumor tissue than in normal tissue (Fig. 4D). Besides, the expression of STIL was significantly distinct among different cell clusters (Fig. 4E).

### Gene mutation profile between STIL-high and STIL-low expression group

**Fig. 5:**
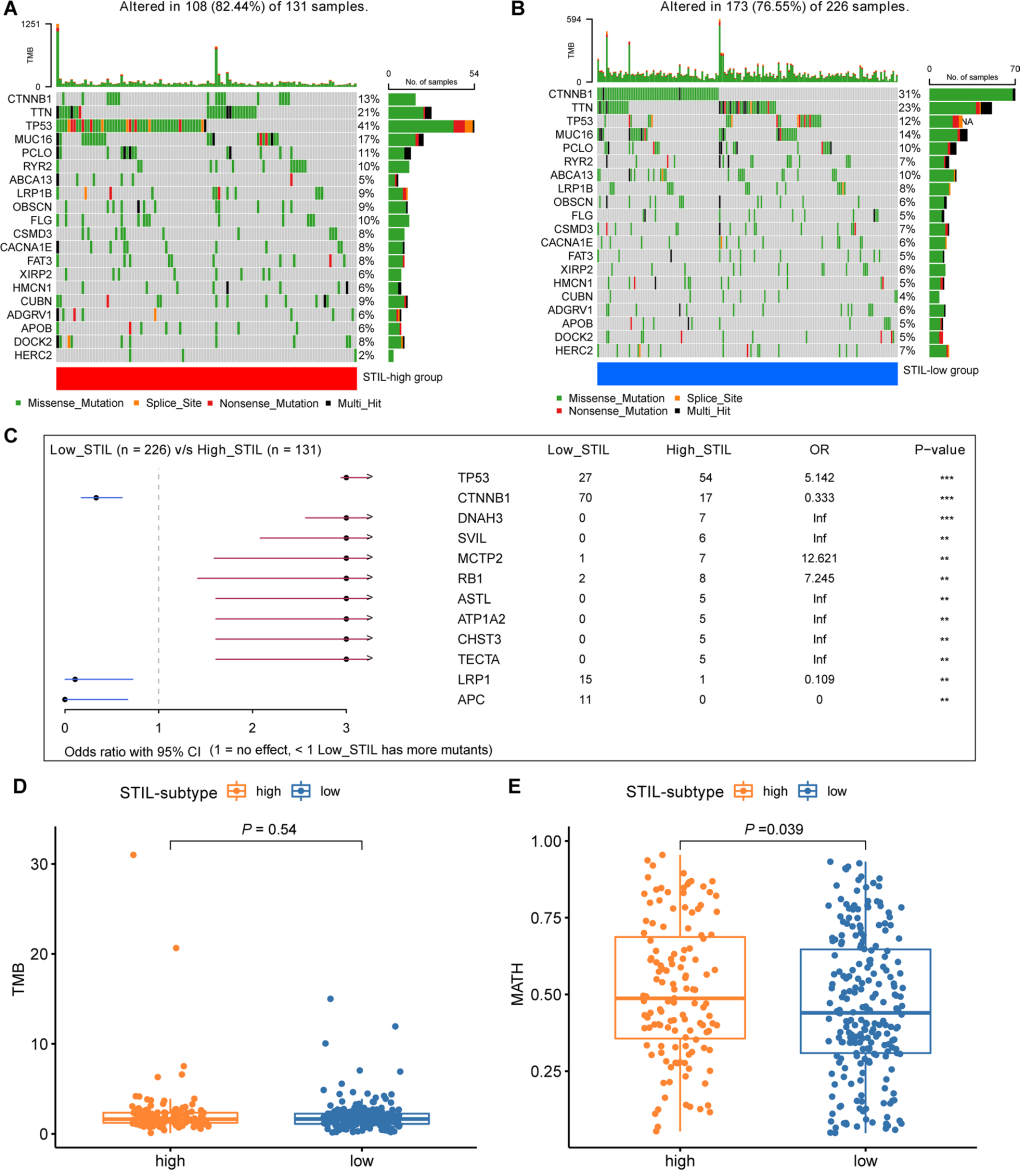
Somatic mutation profiles between STIL-high and STIL-low expression group. Oncoplots exhibited the top-20 mutated genes in the **A** high-STIL and **B** low-STIL subset of the TCGA-LIHC cohort. **C** Forest plot displayed twelve mutated genes with statistic significant between the STIL-high and STIL-low group. The comparison of TMB (**D**) and MATH (**E**) scores in the STIL-high and STIL-low group. ***p < 0.001, **p < 0.01

To probe the genomic alterations between STIL-high and STIL-low subsets, we performed somatic mutation profiles from the TCGA-LIHC datasets. The top-5 highest mutated genes in the STIL-high subset (Fig. 5A) were TP53 (41%), TTN (21%), MUC16 (17%), CTNNB1 (13%), RYR2 (10%), whereas those in the STIL-low subset (Fig. 5B) were CTNNB1 (31%), TTN (23%), MUC16 (14%), TP53 (12%), and PCLO (10%). We found nine genes (TP53, DNAH3, SVIL, MCTP2, RB1, ASTL, ATP1A2, CHST3, and TECTA) have higher mutation prevalence in the STIL-high subset than in the STIL-low subset, while three genes (CTNNB1, LRP1, and APC) displayed highly mutated rates in STIL-low subset (Fig. 5C). Although the TMB was not significantly different between STIL-low and STIL-high subsets (Fig. 5D), patients from high-STIL subset exhibited significantly elevated MATH scores, suggesting a higher level of tumor heterogeneity in this subset (Fig. 5E, P = 0.039).

### Functional analysis of STIL

**Fig. 6:**
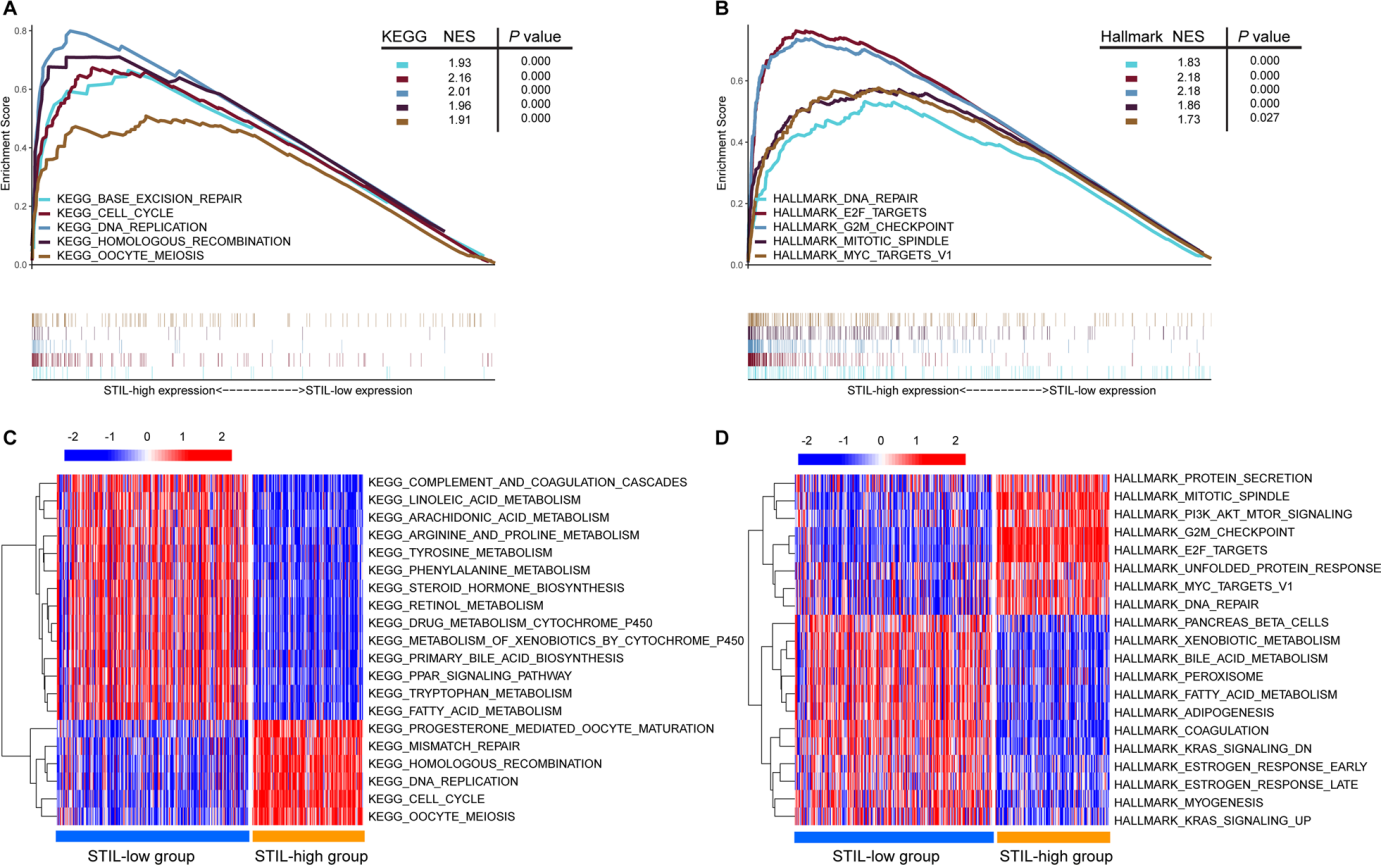
The gene set enrichment analysis (GSEA) and Gene set variation analysis (GSVA) of STIL in the TCGA-LIHC dataset. **A** Top 5 pathways in the high-STIL expression phenotype predicted by GSEA analysis; **B** Top 5 hallmarks in the high-STIL expression phenotype predicted by GSEA analysis; **C** GSVA-derived clustering heatmaps of top 20 differentially expressed pathways for STIL expression; **D** GSVA-derived clustering heatmaps of top 20 differentially expressed hallmarks for STIL expression

We further probed the potential mechanisms of STIL on the influence of survival by performing the GSEA and GSVA. GSEA of the KEGG gene set and Hallmark gene set were performed, and the results revealed that the STIL-high group was mostly enriched in several functional pathways related to tumor proliferation and DNA damage, such as KEGG_CELL_CYCLE, KEGG_BASE_EXCISION_REPAIR, HALLMARK_DNA_REPAIR, and HALLMARK_E2F_TARGETS (Fig. 6A, B). Consistently, GSVA analysis showed similar results that the cell proliferation gene sets (such as KEGG_CELL_CYCLE, KEGG_OOCYTE_MEIOSIS, HALLMARK_MITOTIC_SPINDLE, and HALLMARK_E2F_TARGETS) and DNA damage related sets (such as KEGG_MISMATCH_REPAIR, KEGG_HOMOLOGOUS_RECOMBINATION, and HALLMARK_DNA_REPAIR) were significantly overexpressed in the STIL-high group (Fig. 6C, D). These results suggested that the overexpression phenotype of STIL was positively associated with pathways enriched in cell proliferation and DNA damage response, which were reported to be associated with cancer initiation, progression, and immune response [47–49].

### Construction of ceRNA regulatory network of STIL

**Fig. 7:**
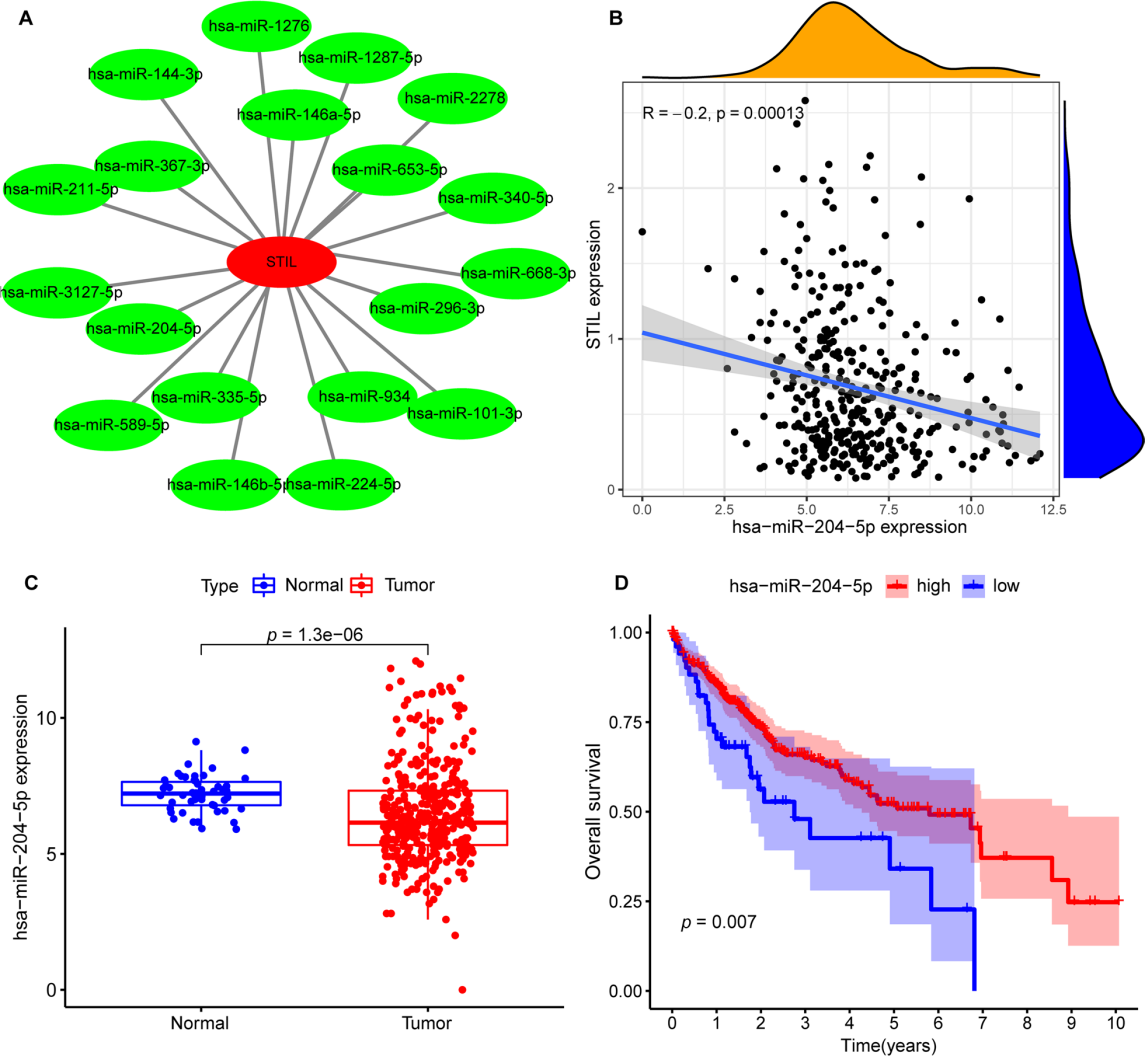
has-miR-204-5p was screened out as a potential upstream miRNA of STIL in HCC. **A** Cytoscape software displayed the miRNAs-STIL regulatory network. **B** The expression relationship between has-miR-204-5p and STIL in HCC. **C** The differential expression of has-miR-204-5p in HCC tissues and normal tissues. **D** Kaplan–Meier (KM) survival analysis of has-miR-204-5p in HCC

Non-coding RNAs (ncRNAs) have been widely acknowledged as the regulator of gene expression. To test whether STIL was regulated by some ncRNAs, we first used the ENCORI, a bioinformatics tool, to screen potentially upstream miRNAs of STIL and finally selected 19 miRNAs. A Cytoscape visualization of the miRNAs-STIL regulatory network was constructed (Fig. 7A). Mechanism of action relied on miRNA indicating that there should be a negative association between miRNA and STIL. We then performed the differential analysis and correlation analysis, and only hsa-miR-204-5p was retained, with a negative correlation of STIL and significantly downregulated expression in tumor tissue than normal tissue (Fig. 7B, C). Finally, we explored the prognostic value of hsa-miR-204-5p, and found that high levels of hsa-miR-204-5p predict a favorable prognosis for HCC (Fig. 7D). Taken together, hsa-miR-204-5p might be the most potential regulatory miRNA of STIL in HCC.

**Fig. 8:**
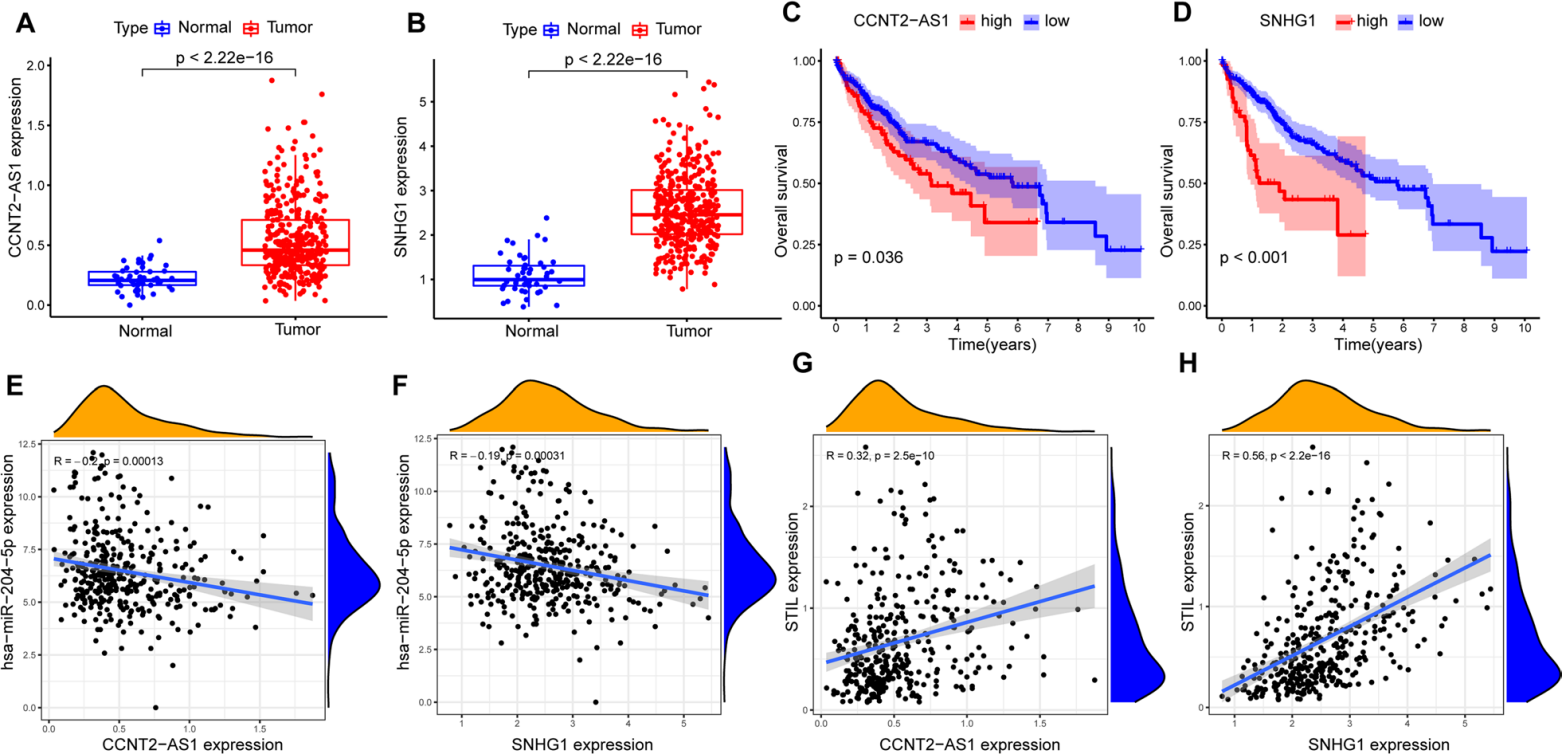
Differential expression analysis, KM survival analysis and Spearman’s correlation analysis. Differential expression analysis showed that CCNT2-AS1 (**A**) and SNHG1 (**B**) expression were significantly higher in tumor tissues than normal tissues; KM survival curves showed that the high expression of CCNT2-AS1 (**C**) and SNHG1 (**D**) exhibited a poor survival. The has-miR-204-5p expression possessed a significantly negative relationship with CCNT2-AS1 (**E**) and SNHG1 (**F**); The STIL expression possessed a significantly positive relationship with CCNT2-AS1 (**G**) and SNHG1 (**H**)

**Fig. 9:**
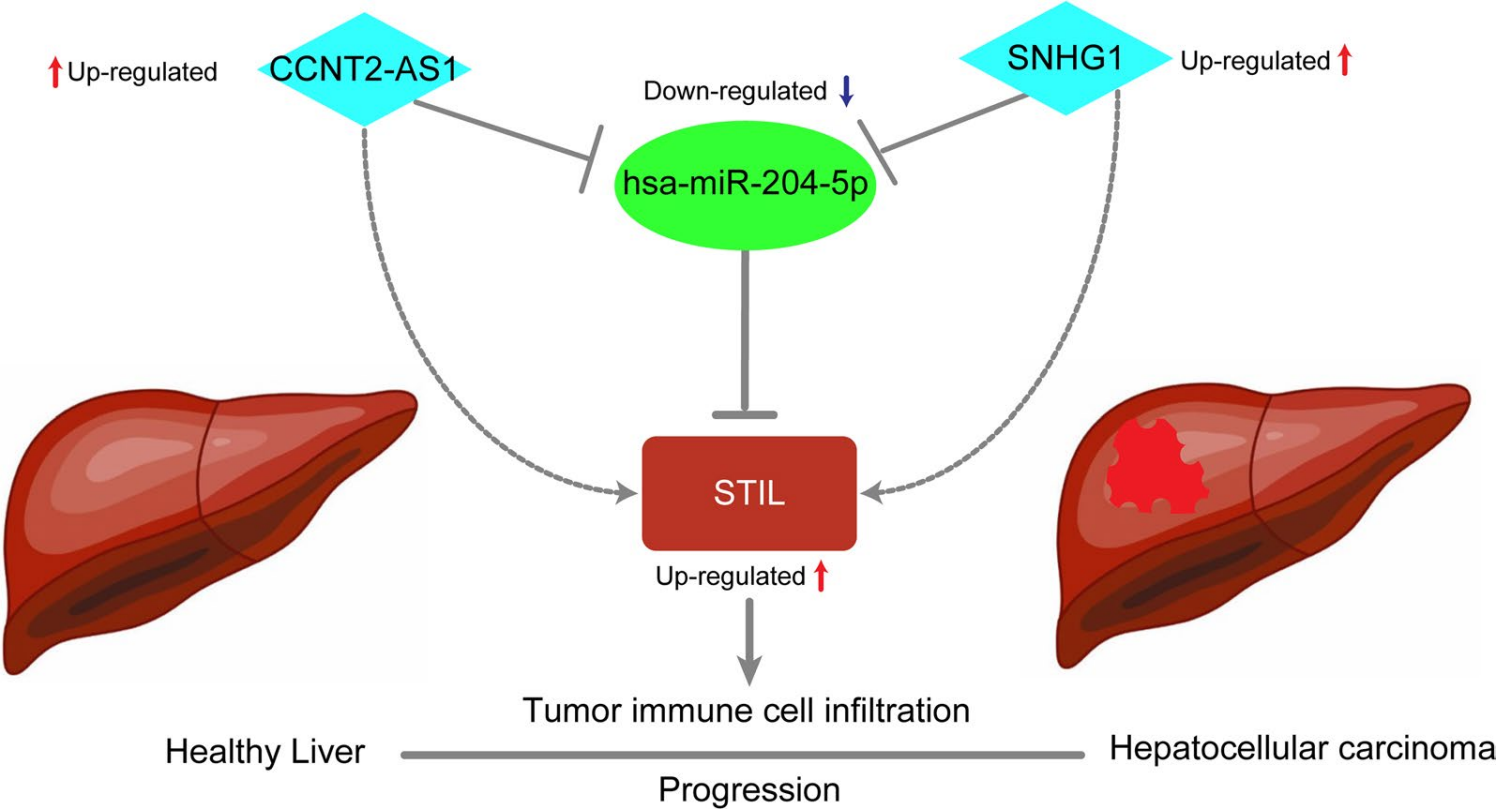
The model of CCNT2-AS1/SNHG1- has-miR-204-5p -STIL axis in carcinogenesis of HCC

Next, the 119 upstream lncRNAs of hsa-miR-204-5p were predicted respectively using ENCORI database (Additional file 1: Table S1). Then, the expression analysis, survival analysis, and correlation analysis were determined using the Wilcoxon test, log-rank test, and Spearman's correlation analysis, respectively, in R software. We considered the most potential upstream lncRNAs of has-miR-204-5p should meet the following criteria simultaneously. First, the expression of these lncRNAs was overexpressed in tumor tissue than normal tissue with P-value < 0.001 (Fig. 8A, B); Second, these lncRNAs were significantly associated with prognosis (Fig. 8C, D); Third, based on the ceRNA hypothesis, these lncRNAs should be a negative relationship with hsa-miR-204-5p and positive correlation with STIL (Fig. 8E–H). Last, there were two lncRNAs (CCNT2-AS1 and SNHG1) with the most potential upstream lncRNAs of hsa-miR-204-5p retained in this study, and a lncRNA-miRNA-mRNA regulatory network was constructed (Fig. 9).

### STIL expression correlates with the infiltration levels of immune cells in HCC

**Fig. 10:**
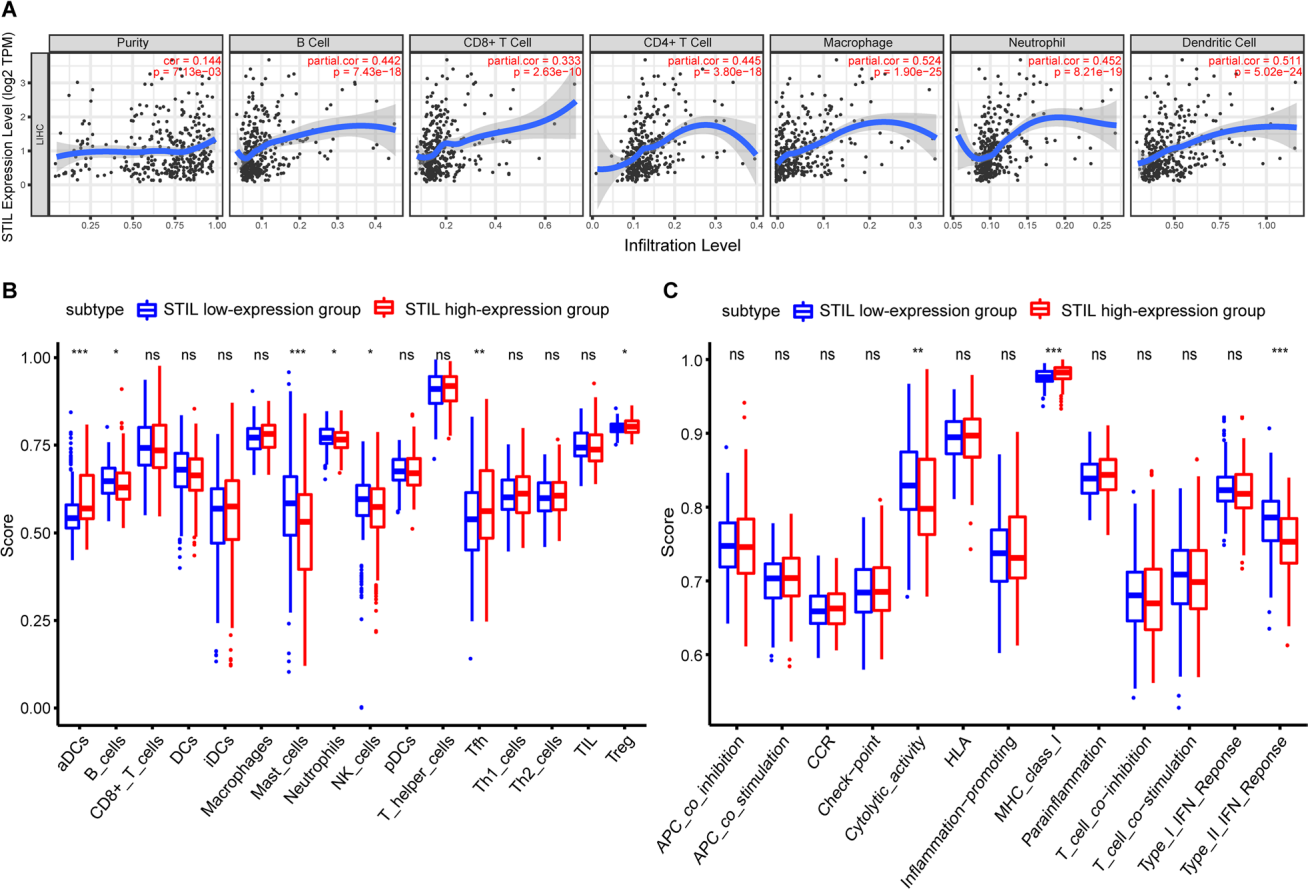
Association between STIL expression and tumor-infiltrating immune cells (TIICs) and related functions or pathways in HCC.** A** Exploiting the TIMER database, the relationship between STIL and the infiltration levels of B cells, CD8 + T cells, CD4 + T cells, macrophages, neutrophils and dendritic cells was displayed in scatterplots based on the purity-corrected Spearman method; **B**, **C** Comparison of the ssGSEA scores between different STIL expression groups in the TCGA cohort

Dysregulated STIL is exert a crucial function in chromosomal instability which is found to be involved in the tumor immune microenvironment. Besides, tumor-infiltrating immune cells have a huge effect on the clinical outcomes of patients with multiple cancers [50, 51]. Therefore, we investigated the association between STIL expression and the tumor-infiltrating immune cells in HCC using the TIMER database. We observed that the levels of STIL expression were positively associated with tumor purity (cor = 0.144, P = 7.13e−03), indicating STIL expression was found mainly from the tumor cells. Moreover, the expression levels of STIL were positively associated with the infiltration levels of B cells (r = 0.442, *P* = 7.43e−18), CD8+ T cells (r = 0.333, *P* = 2.63e−10), CD4 + T cells (r = 0.445, *P* = 3.80e−18), Macrophages (r = 0.524, *P* = 1.90e−25), Neutrophils (r = 0.452, *P* = 8.21e−19), DCs (*r* = 0.511, P = 5.02e−24) in HCC tissues (Fig. 10A). These findings indicated that STIL is closely correlated with the levels of immune cells in HCC.

To further probe the relationship between the STIL expression and immune status, we computed the ES (enrichment scores) of sixteen immune cells and thirteen immune-related pathways and functions with ssGSEA. Interestingly, the high-STIL expression group has a higher level of scores in aDCs (activated dendritic cells) and MHC class I, which both related to the antigen presentation process, than the low-STIL expression group in the TCGA cohort (adjusted *P* < 0.05, Fig. 10B). Moreover, the score levels of type II IFN response, Neutrophils, B cells, NK cells, and Cytolytic activity were lower in the high-STIL expression group, while the activity of Tregs and Tfh was just the opposite (adjusted *P* < 0.05, Fig. 10C).

### Correlation analysis between STIL and biomarkers of potential immunotherapy targets

**Fig. 11:**
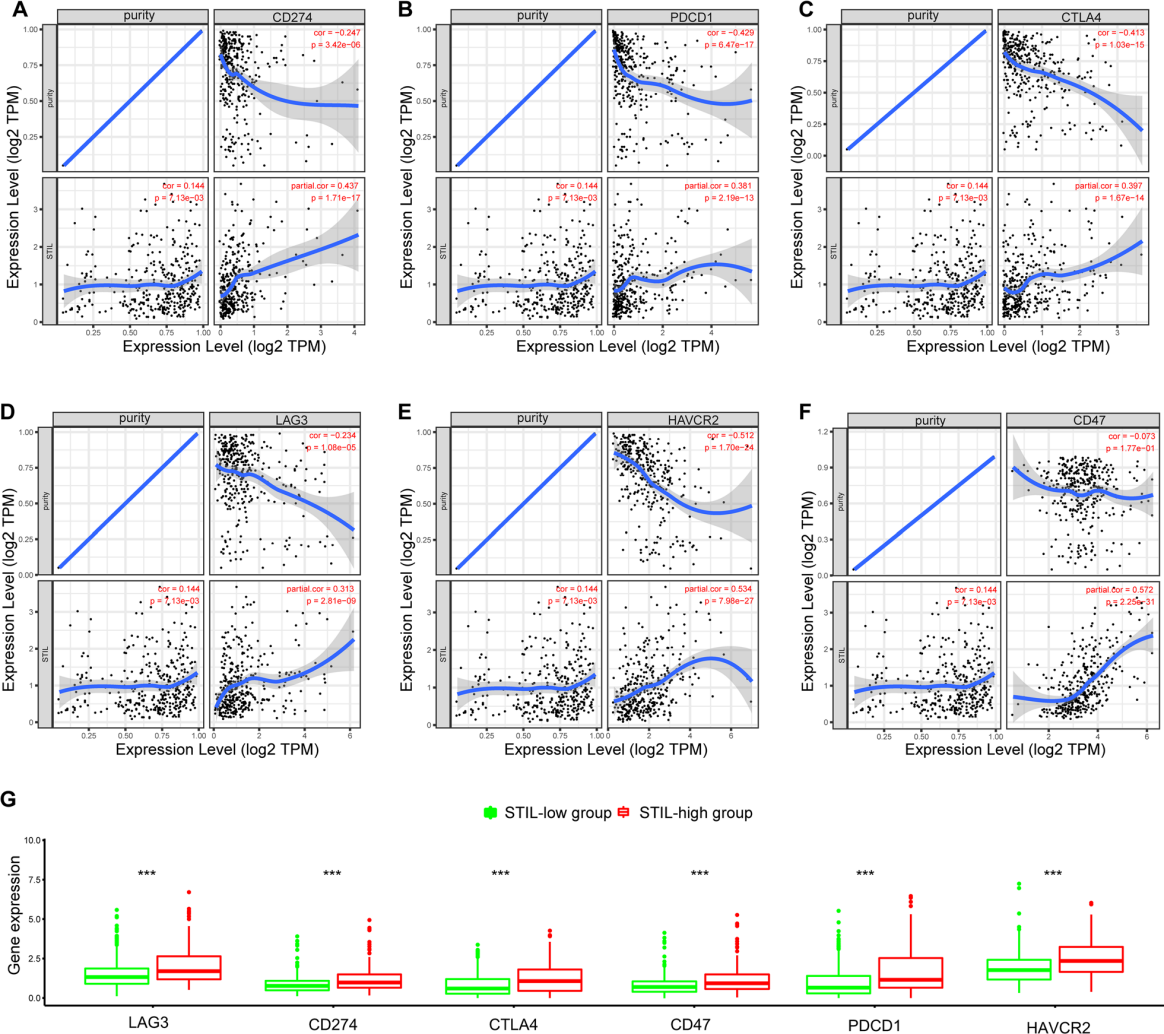
Relationship between the expression of STIL and several promising immunotherapeutic targets via the TIMER database. **A**–**F** The scatterplots of correlation between STIL expression and the promising immunotherapeutic targets (CD274, PDCD1, CTLA-4, LAG3, HAVCR2, and CD47. **G** The levels of promising immunotherapeutic targets between the high- and low-STIL expression groups

The inhibitory molecules-targeted immunotherapies, such as anti-CTLA-4, -PD-1/L1, and -CD47 inhibitors, have offered new opportunities for treating patients with hard-to-treat malignancies and thus improving their survival rates [52–54]. We then assessed the relationship between STIL and multiple promising immunotherapy targets, including LAG3, CD274 (PD-L1), CTLA4, CD47, PDCD1 (PD-1), and HAVCR2 (TIM-3), and results showed STIL was positively associated with these immunotherapy targets (Fig. 11A–F). Moreover, these immunotherapy targets were significantly upregulated in the high-STIL expression group (Fig. 11G).

### STIL predicts immunotherapy/chemotherapy efficacy

**Fig. 12:**
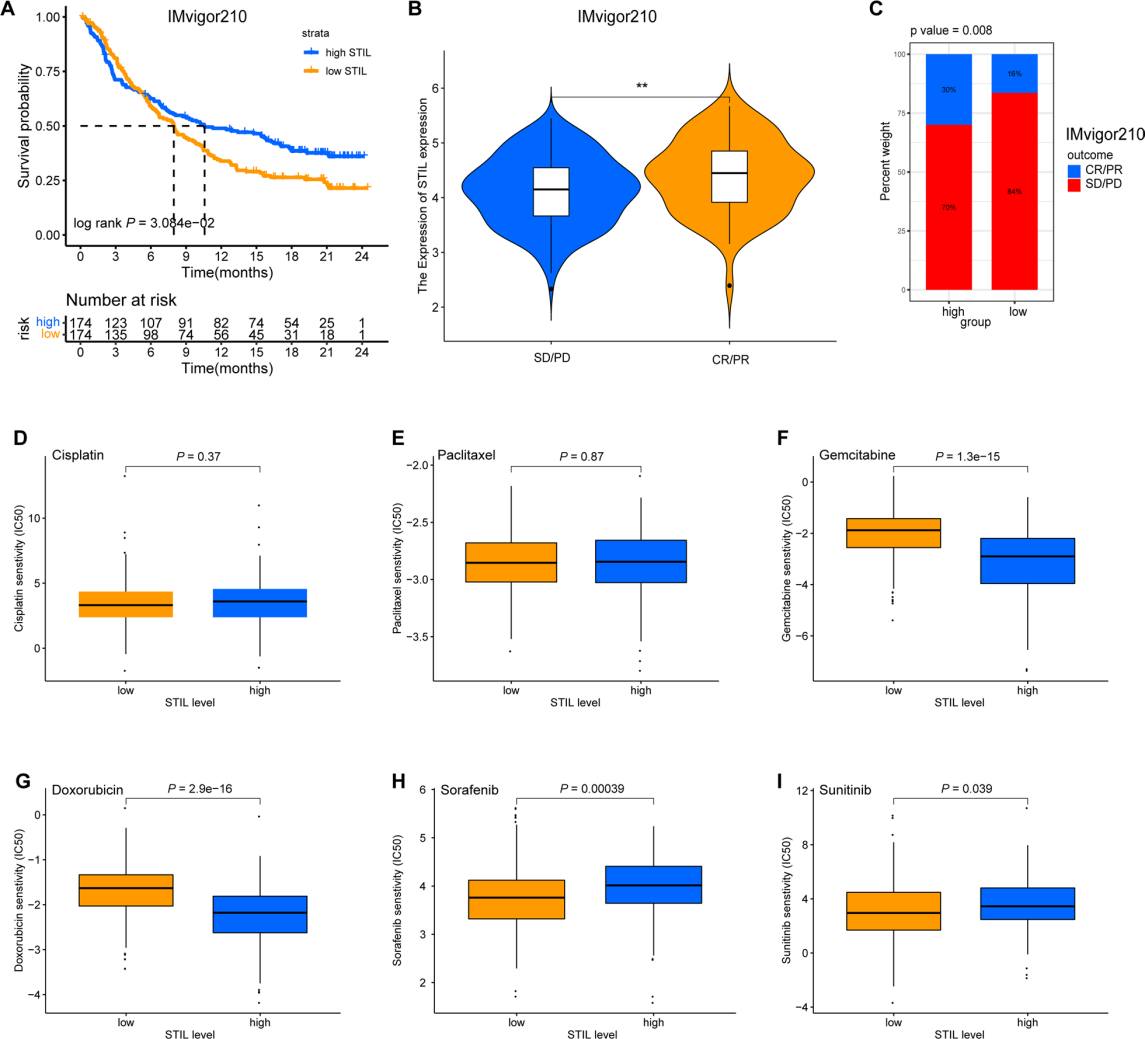
The expression level of STIL can predict immunotherapy/chemotherapy efficacy.** A** KM survival curves showed that patients with high-STIL expression have a favorable prognosis than those with low-STIL expression in IMvigor 210 cohort. **B** The levels of STIL expression were grouped by immunotherapy efficacy. **C** Comparison of immunotherapy efficacy between the high- and low-STIL expression groups. **D**–**I** The comparison of the IC50 values of six common chemotherapeutic drugs between the high- and low-STIL expression groups

The strong positive relationship between STIL and immune cell infiltrations and multiple promising immunotherapy molecules that were overexpressed in patients with high-STIL expression prompted us to address whether the STIL expression level predicts immunotherapy efficacy. An immunotherapy cohort (IMvigor210 cohort) integrating the therapeutic effects of patients who underwent immunotherapy (anti-PD-L1) and transcriptome data was utilized to explore the prognostic value of the STIL expression for immune blockade therapy. Patients with high-STIL expression were associated with a more favorable prognosis than those with low-STIL expression (Fig. 12A). The complete/partial response (CR/PR) has a higher STIL expression than the SD/PD efficacy subgroup after dividing the immunotherapeutic efficacy in a binary mode (Fig. 12B). We also found that the high-STIL expression group has a higher percentage of CR/PR than the low-STIL group (Fig. 12C). Moreover, the treatment-related IC50 of six agents (including cisplatin, paclitaxel, gemcitabine, doxorubicin, sorafenib, and sunitinib) in patients from TCGA-LIHC cohort was performed to evaluate the sensitivity of the two risk subsets to these drugs, and we observed that STIL-high patients were more sensitive to the gemcitabine, doxorubicin, while more resistant to the sorafenib and sunitinib (Fig. 12D–I).

### STIL knockdown inhibits HCC growth, invasion, and migration

**Fig. 13:**
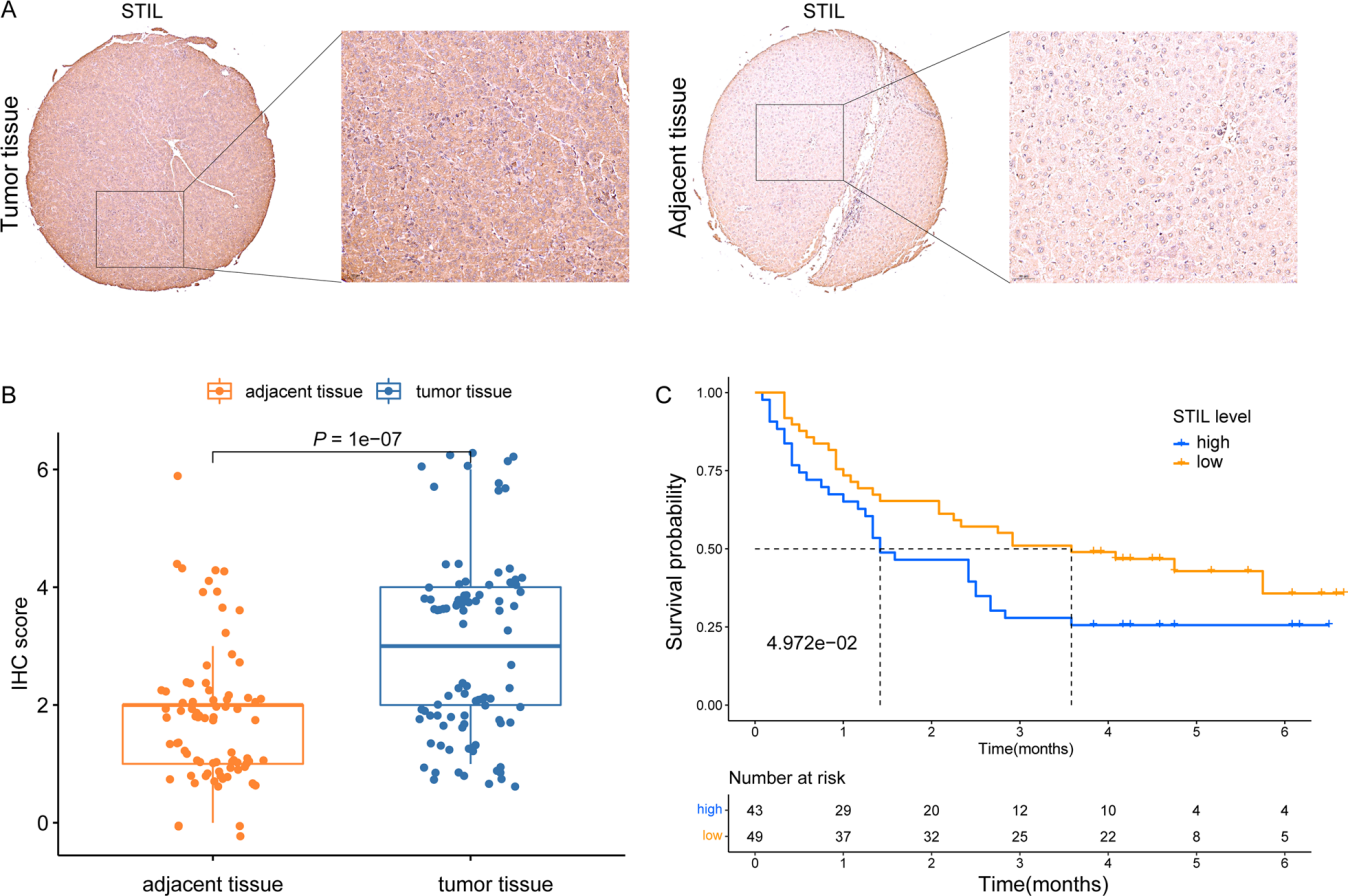
The expression of STIL in HCC tissues and its prognostic significance were assessed by immunohistochemistry. **A** Representative IHC image of STIL expression in tumor and adjacent tissues. **B** showed that the expression of STIL is distinct between tumor tissues and adjacent liver tissues. **C** High STIL expression in HCC was associated with poor clinical prognosis

**Fig. 14:**
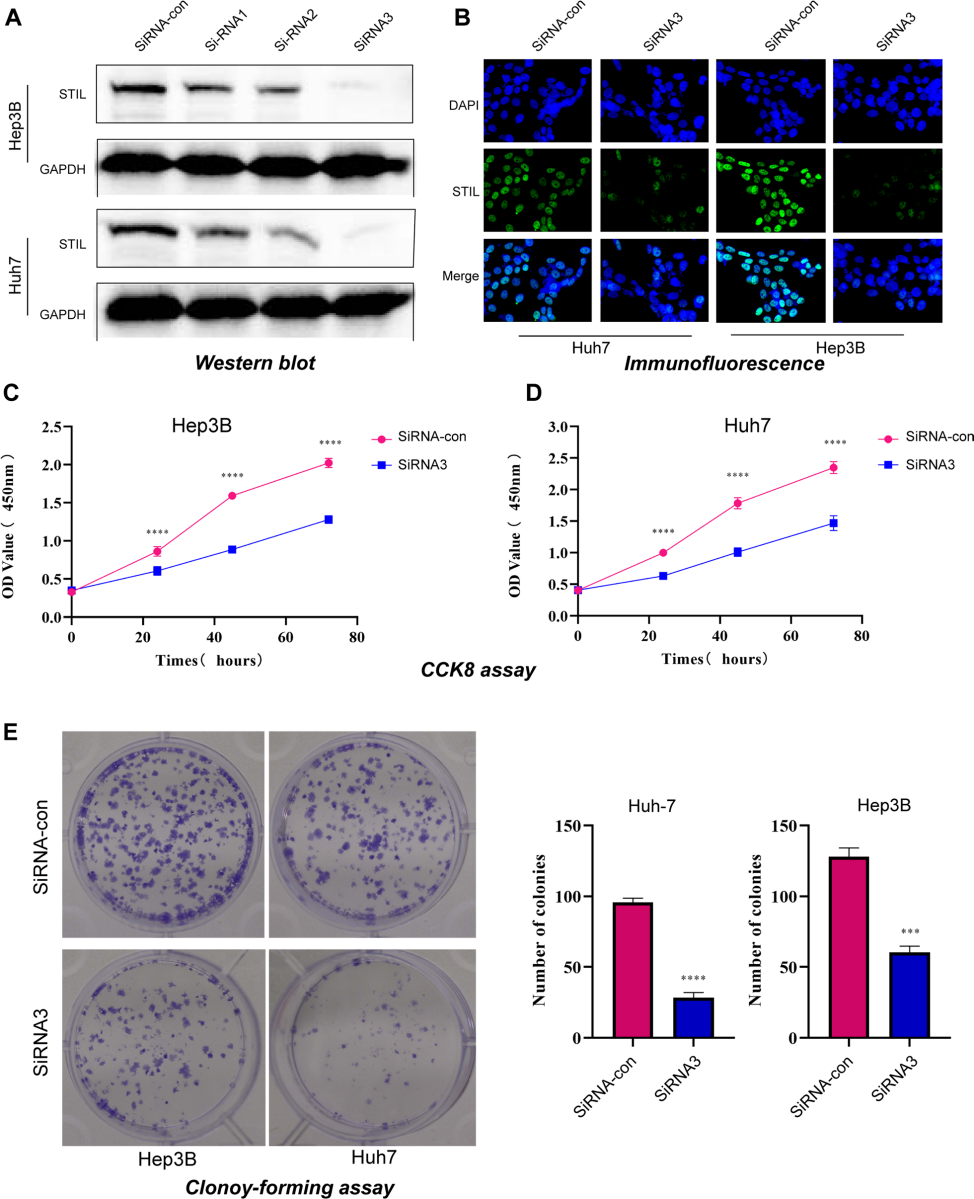
STIL-silencing impeded HCC cell proliferation properties in vitro. **A** Expression levels of STIL were determined by western blot in HCC cells treated with siRNA-STIL and siRNA-con. STIL knockout hindered HCC cell growth assessed by immunofluorescence assay (**B**), CCK-8 assay (**C**, **D**) and colony-forming assay (**D**)

**Fig. 15:**
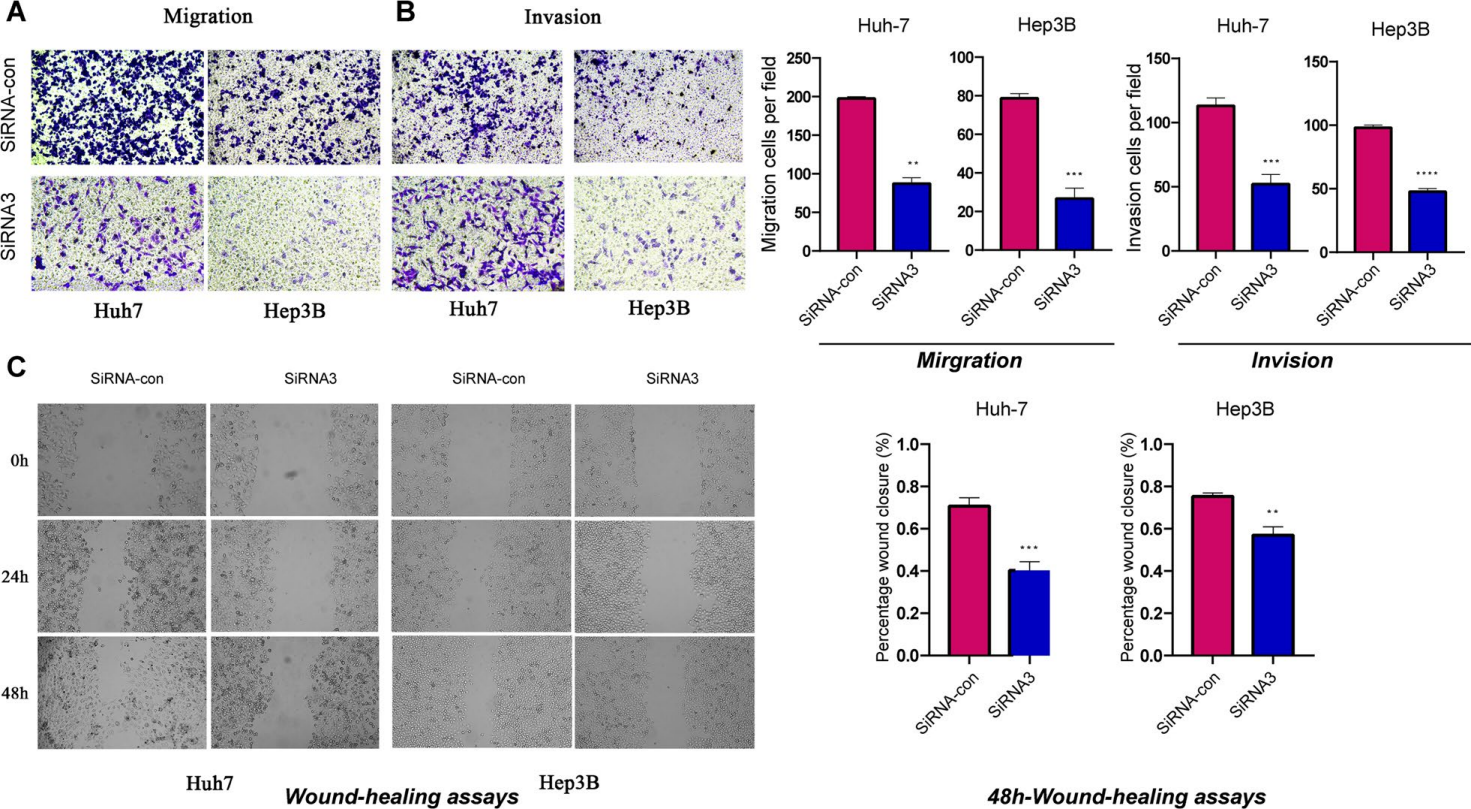
STIL-silencing blocked HCC cell migration and invasion in vitro. **A**, **B** Representative images and computed of the number of cells that migrated or invaded in transfected HCC cell lines. Experiments were repeated three times with similar results, and error bars represent the mean ± SEM, ***p < 0.001 **p < 0.01. **C** Wound-healing assays were used to detect the migration ability of transfected cells

We first performed an immunohistochemistry assay with STIL antibody to validate our findings, using a tissue microarray (TMA). Figure 13A showed the representative image of STIL expression in the tumor and adjacent tissues. In differential analysis, the expression of STIL protein was significantly upregulated in tumor tissue compared with adjacent normal tissue (Fig. 13B). Survival analysis suggested that patients with high-STIL expression were associated with poor prognosis (Fig. 13C). We then investigate the biological effects of siRNA-induced knockdown of STIL expression on HCC cell lines. Knockdown of STIL in HCC cell lines, as verified by western blot assay (Fig. 14A, Additional file 2: Figure S1), significantly reduced the proliferation of HCC cells, using Immunofluorescence assay, CCK8 assay, and colony-forming assay (Fig. 14B–E). Evaluation of the impacts of STIL knockdown on the invasion and migration ability of HCC cells, as determined by transwell (Fig. 15A, B) and wound healing assays (Fig. 15C), showed that knockdown of STIL inhibited cell invasion and migration. Collectively, these results showed that STIL was highly expressed in tumor tissue, high-STIL expression predicts poor OS, and was significantly associated with HCC cell proliferation, invasion, and migration.

## Discussion

In this study, our findings demonstrated that STIL is a promising independent prognostic indicator and also a biomarker for the therapeutic efficacy of HCC. GSEA and GSVA reveal that the high expression phenotype of STIL in HCC may likely have far-reaching influences on the cell cycle and genomic stability. Also, the expression levels of STIL were positively associated with the tumor-infiltrating immune cells and its marker genes in HCC. Interestingly, the upregulated STIL expression was associated with marker genes of T regulatory cells, exhaustion-associated inhibitory receptors, and the benefit of chemotherapeutic drugs and PD-L1-targeted immunotherapy, implying a potential novel immune regulatory role of STIL in immune escape and thus exerts a huge impact on immunotherapeutic efficacy.

STIL level in lung and ovarian cancers and nasopharyngeal and prostate carcinoma was reported to be higher in tumor tissues than in adjacent normal tissues and was correlated with higher metastatic potential in various cancers [15, 25, 27, 55]. However, there exists no systematic and elaborated report on the expression of STIL in HCC tissue and its relationship with the clinical outcomes of patients. Thus, in our study, we found that STIL expression was significantly higher in hepatocellular carcinoma tissue than in normal liver tissue in HCCDB and Oncomine databases, The IHC analysis of tissue microarray (TMA) and scRNA-seq data confirmed the above results. Besides, elevated STIL was significantly correlated with aggressive factors, such as late-stage, age, grade, T stage, and AFP. Furthermore, high STIL expression was independently associated with poor overall survival in the TCGA cohort. Upregulation of STIL still predicted decreased overall survival in the ICGC-LIRI-JP and TMA cohorts. When combined with other clinical factors, such as age, gender, grade, stage, T stage, N stage, and M stage, high expression of STIL remains to indicate a poor outcome in the TCGA-LIHC cohort, using univariate and multivariate analyses. Moreover, we also evaluated the effect of STIL on the proliferation, invasion, and migration of HCC cells. We found that the knockout of STIL impeded the growth of HCC cells by fluorescence assay, CCK8 assay, and colony formation assay, and inhibited cell invasion and migration by transwell and wound healing assays. Function enrichment analysis of GSEA and GSVA revealed that STIL was mainly correlated with the cell proliferation-related pathways (such as Cell cycle, E2F-targets, and Mitotic spindle) but also with pathways referring to DNA damage response that affected gene instability, including DNA replication and Mismatch repair in HCC. Thus, we have reason to believe that STIL functions as an oncogene and is a potential prognostic indicator for the survival of HCC.

The role of non-coding RNAs (including miRNAs, lncRNAs, and circular RNAs) in regulating mRNA gene expression has been well documented. To explore the molecular regulatory mechanisms operating upstream of the STIL expression, multiple miRNA-target prediction programs using the ENCORI database (The Encyclopedia of RNA Interactomes) were performed, and has-miR-204-5p was screened out after performing correlation analysis, expression analysis, and survival analysis. Previous studies showed that has-miR-204-5p exerted inhibitory roles in tumor cell proliferation, migration, and metastasis [56, 57], indicating it may be a tumor suppressor in HCC. Subsequently, a ceRNA regulatory network was screened based on the has-miR-204-5p and STIL. By performing expression analysis, survival analysis, and correlation analysis, two lncRNAs (CCNT2-AS1 and SNHG1) were finally selected as the most potential upregulated lncRNAs. SNHG1 has been found overexpressed in HCC tissue and is significantly associated with decreased survival of HCC [58]. Moreover, overexpressed SNHG1 acts a crucial function in cancer progression, invasion, and metastasis in multiple malignancies, including HCC [59–62]. The study of CCNT2-AS1 has not yet been reported, which needs further explored its role in HCC. Collectively, CCNT2-AS1 and SNHG1/ has-miR-204-5p/STIL axis were identified as potential regulatory pathways in HCC.

Recent studies showed that the integration of TIICs (Tumor-infiltrating immune cells) and clinicopathologic factors could serve as a prognostic, predictive signature and predict the therapeutic response [63, 64]. This prompted us to decipher whether there is a relationship between STIL expression and TIICs. Our current findings revealed that the expression level of STIL was correlated with diverse immune cell types in HCC tissues. Specifically, we observed a positive relationship between STIL expression and infiltrating levels of B cells, CD8+ T cells, CD4+ T cells, macrophages, neutrophils, and dendritic cells. We also found that the high-STIL expression group has a higher level of scores in aDCs and MHC class I, which are both associated with the antigen presentation process, than the low-STIL expression group. Besides, a higher fraction of Treg cells was observed in the high-STIL expression group. Previous studies have demonstrated that Treg cells [65] are linked with clinical poor outcomes for HCC patients because of their role in immune escape. Moreover, the STIL high-expression group undermined antitumor immunity, including the function of type II IFN response and the abundance of NK cells. In addition, although TMB was not distinguish between the STIL-high subset and STIL-low subset, Patients with high STIL expression exhibited higher MATH scores, an indicator for tumor heterogeneity, which is generally associated with impaired immunity in multiple malignancies [66]. Therefore, these data suggest that high STIL expression levels might be correlated with immunosuppression in HCC.

Strategies for targeting Immune checkpoint-related genes have been proven to improve response rates of anti-PD-1/PD-L1 therapy [67, 68]. For instance, TIM3 inhibitor co-blockade with PD-1 restores the cytotoxic function of CD8+ T cells, indicating the potential of using combinations of anti-PD-1/PD-L1 therapy. Besides, combination therapy against different targets showed promising improved immunotherapy response rates, and a recent study showed that a combination of atezolizumab (an anti-PD-L1 inhibitor) with bevacizumab (an anti-VEGFR inhibitor) led to enhancing antitumor activity and safety for patients with unresectable hepatocellular carcinoma [69]. In the present study, we demonstrated that STIL expression was significantly associated with tumor immune infiltrations and promising immunotherapy targets (such as PD-L1, CTLA-4, and TIM3), indicating that targeting STIL might increase the efficacy of immunotherapy in HCC. Moreover, we also observed that patients with high-STIL expression show the better benefit of an immune checkpoint inhibitor (ICI) in the IMvigor210 cohort and two chemotherapeutic drugs (gemcitabine and doxorubicin). Therefore, it is tempting to speculate that STIL may have a regulatory role in tumor immunity and could be part of combination therapy approaches for enhancing the responsiveness to immune checkpoint inhibitors.

Some limitations in the present study should be recognized. First, the ceRNA regulation axis of STIL was only predicted using online databases, thereby needing to be validated in vitro experiments; second, the potential molecular mechanisms of STIL and tumor-immune cell interactions in HCC should be confirmed in vivo experiments. Third, the clinical profiles on the immunotherapy for HCC patients were undocumented, which hindered a more specific insight from being conducted.

In summary, our findings reveal that STIL is a promising independent prognostic indicator and a biomarker for the immunotherapy benefit of HCC. Additionally, we found several pathways linked with STIL in HCC, such as cell cycle, DNA replication, oocyte meiosis, mismatch repair, homologous recombination, ubiquitin-mediated proteolysis, and the base excision repair pathway. We also identified an upstream regulatory molecular mechanism of STIL in HCC, CCNT2-AS1/SNHG1-has-miR-204-5p-STIL axis. Importantly, STIL expression is strongly related to the infiltration of immune cells, the expression of several immune checkpoints, and the survival benefit of ICI treatment and chemotherapeutic drugs, shedding light on further exploring novel immune-based combination therapies for cancers.

## Supplementary Information

**Additional file 1: Table S1.** The predicted upstream lncRNAs of hsa-miR-204-5p using the ENCORI database.

**Additional file 2: Figure S1.** The raw figure of figure 14A processed by esm.

## Data Availability

The datasets used and/or analyzed during the present study are available from the corresponding author upon reasonable request.
